# Assessment of Facial Morphologic Features in Patients With Congenital Adrenal Hyperplasia Using Deep Learning

**DOI:** 10.1001/jamanetworkopen.2020.22199

**Published:** 2020-11-18

**Authors:** Wael AbdAlmageed, Hengameh Mirzaalian, Xiao Guo, Linda M. Randolph, Veeraya K. Tanawattanacharoen, Mitchell E. Geffner, Heather M. Ross, Mimi S. Kim

**Affiliations:** 1 Information Sciences Institute, University of Southern California, Los Angeles; 2 Department of Electrical and Computer Engineering, University of Southern California, Los Angeles; 3 Division of Medical Genetics, Children’s Hospital Los Angeles, Los Angeles, California; 4 Keck School of Medicine of the University of Southern California, Los Angeles; 5 Center for Endocrinology, Diabetes, and Metabolism, Children’s Hospital Los Angeles, Los Angeles, California; 6 The Saban Research Institute at Children’s Hospital Los Angeles, Los Angeles, California

## Abstract

**Importance:**

Congenital adrenal hyperplasia (CAH) is the most common primary adrenal insufficiency in children, involving excess androgens secondary to disrupted steroidogenesis as early as the seventh gestational week of life. Although structural brain abnormalities are seen in CAH, little is known about facial morphology.

**Objective:**

To investigate differences in facial morphologic features between patients with CAH and control individuals with use of machine learning.

**Design, Setting, and Participants:**

This cross-sectional study was performed at a pediatric tertiary center in Southern California, from November 2017 to December 2019. Patients younger than 30 years with a biochemical diagnosis of classical CAH due to 21-hydroxylase deficiency and otherwise healthy controls were recruited from the clinic, and face images were acquired. Additional controls were selected from public face image data sets.

**Main Outcomes and Measures:**

The main outcome was prediction of CAH, as performed by machine learning (linear discriminant analysis, random forests, deep neural networks). Handcrafted features and learned representations were studied for CAH score prediction, and deformation analysis of facial landmarks and regionwise analyses were performed. A 6-fold cross-validation strategy was used to avoid overfitting and bias.

**Results:**

The study included 102 patients with CAH (62 [60.8%] female; mean [SD] age, 11.6 [7.1] years) and 59 controls (30 [50.8%] female; mean [SD] age, 9.0 [5.2] years) from the clinic and 85 controls (48 [60%] female; age, <29 years) from face databases. With use of deep neural networks, a mean (SD) AUC of 92% (3%) was found for accurately predicting CAH over 6 folds. With use of classical machine learning and handcrafted facial features, mean (SD) AUCs of 86% (5%) in linear discriminant analysis and 83% (3%) in random forests were obtained for predicting CAH over 6 folds. There was a deviation of facial features between groups using deformation fields generated from facial landmark templates. Regionwise analysis and class activation maps (deep learning of regions) revealed that the nose and upper face were most contributory (mean [SD] AUC: 69% [17%] and 71% [13%], respectively).

**Conclusions and Relevance:**

The findings suggest that facial morphologic features in patients with CAH is distinct and that deep learning can discover subtle facial features to predict CAH. Longitudinal study of facial morphology as a phenotypic biomarker may help expand understanding of adverse lifespan outcomes for patients with CAH.

## Key Points

**Question:**

Do patients with congenital adrenal hyperplasia (CAH) have distinct facial morphologic features that are distinguishable by deep learning?

**Findings:**

In this cross-sectional study of 102 patients with CAH and 144 control participants, deep learning methods achieved a mean area under the receiver operating characteristic curve of 92% for predicting CAH from facial images. Facial features distinguished patients with CAH from controls, and analyses of facial regions found that the nose and upper face were most contributory.

**Meaning:**

The findings suggest that facial morphologic features, as analyzed by deep neural network techniques, can be used as a phenotypic biomarker to predict CAH.

## Teaser

This cross-sectional study evaluates the use of machine learning for prediction of congenital adrenal hyperplasia based on distinct facial morphologic features.

## Introduction

Congenital adrenal hyperplasia (CAH) due to 21-hydroxylase deficiency is an inherited disorder affecting 1 in 15 000 in the severe, classical form and 1 in 1000 in the mild, nonclassical form.^1^ Congenital adrenal hyperplasia is the most common primary adrenal insufficiency in children, with morbidity and mortality related to life-threatening adrenal crises. Among patients with classical CAH, two-thirds have the salt-wasting form and one-third have the simple-virilizing or non–salt-wasting form. Congenital adrenal hyperplasia is also a disorder of androgen excess, with androgen overproduction from the adrenal glands beginning in week 7 of fetal life, secondary to disrupted steroid biosynthesis.^2^ This excess prenatal androgen exposure and cortisol deficiency could represent a significant change to the intrauterine environment during early development that could adversely program the fetus with CAH for postnatal diseases.

The effects of excess androgens in utero can be readily seen in female newborns with CAH as virilized external genitalia.^3^ Females with CAH also exhibit masculinization of childhood behaviors, including male-typical play preferences, aggression, and altered cognition (eg, spatial ability).^4,5,6,7,8^ Concerning adverse neuropsychological outcomes have also been identified over the lifespan of patients with CAH, including a heightened potential for psychiatric disorders, substance abuse, and suicide,^9,10^ and brain structural abnormalities have been identified in youths and adults with CAH (eg, smaller intracranial volume and smaller regions of the prefrontal cortex and medial temporal lobe).^11,12,13,14^ The association between these outcomes and prenatal hormone abnormalities remains unclear, with a lack of a robust modeling system and a set of biomarkers. The female external genitalia phenotype is scored on a 5-point Prader scale but can vary among patients with a similar genotype.^15^ Amniocentesis to examine prenatal hormones is invasive and not readily available.

This lack of robust phenotypic biomarkers leads us to consider the human face, which contains a wealth of information, including health status and differences by sex.^16,17,18^ Brain and facial morphologic features have been linked in conditions such as fetal alcohol syndrome, although to our knowledge, there is little known about the facial phenotype of patients with CAH.^19^ Sex hormones (ie, testosterone, estrogen) influence the development of sexually dimorphic facial features, with differential morphologic features in adults associated with umbilical cord blood testosterone levels.^20^ Sex differences of the face are evident in childhood and increase during puberty, leading to clear differences in features by adulthood.^21^ Earlier facial analyses have relied on sets of manually engineered features, such as facial width-to-height ratio, masculinity index, or Euclidean distances between facial landmarks.^20,22,23,24,25,26^ However, these techniques have been widely applied to analyze syndromic genetic conditions that have easily recognizable effects on facial morphologic features compared with the more subtle facial features of patients with CAH.^27,28,29,30,31^

Recent advances in deep neural networks have shown promise in analyzing and modeling human faces.^32,33^ Deep learning has revolutionized facial analysis problems, such as age estimation, emotion recognition, and person verification.^34,35,36,37^ Deep networks could be leveraged to detect influence of hormone abnormalities on the facial features of patients CAH. In this study, we examined facial features that could distinguish patients with classical CAH from unaffected, age-matched control individuals, applying facial image analyses that included deep networks and classical machine learning techniques. We hypothesized that facial features would differ between patients with CAH and controls.

## Methods

This cross-sectional observational study was performed at a pediatric tertiary center from November 2017 to December 2019. Patients with classical CAH due to 21-hydroxylase deficiency were recruited at a CAH comprehensive care center in Southern California, over a 2-year period by consecutive sampling. Inclusion criteria were a biochemical diagnosis of salt-wasting or simple-virilizing CAH and age less than 30 years. Healthy, unaffected controls with no serious medical illness were recruited at the hospital pediatric clinics by consecutive sampling. Hispanic ethnicity was classified by the investigators. Tanner staging for puberty (stage I, prepubertal; stage II, pubertal; progression through stages III to V, adult)^38^ was performed for patients with CAH (by endocrinologists [M.E.G., M.S.K.]). We acquired frontal images of the face from patients with CAH and controls using an iPad, version 12.1 (Apple Inc) under normal clinic lighting conditions. We also used convenience sampling to augment the data set with controls selected from 3 publicly available data sets composed of approximately 4 million face images.^39,40,41^ The research protocol was approved by the Children’s Hospital Los Angeles institutional review board. Parents and participants gave written informed assent and consent, respectively, in accordance with the World Medical Association Declaration of Helsinki.^42^ This study followed the Standards for Reporting of Diagnostic Accuracy (STARD) reporting guideline.

### Image Preprocessing

**Figure 1.**
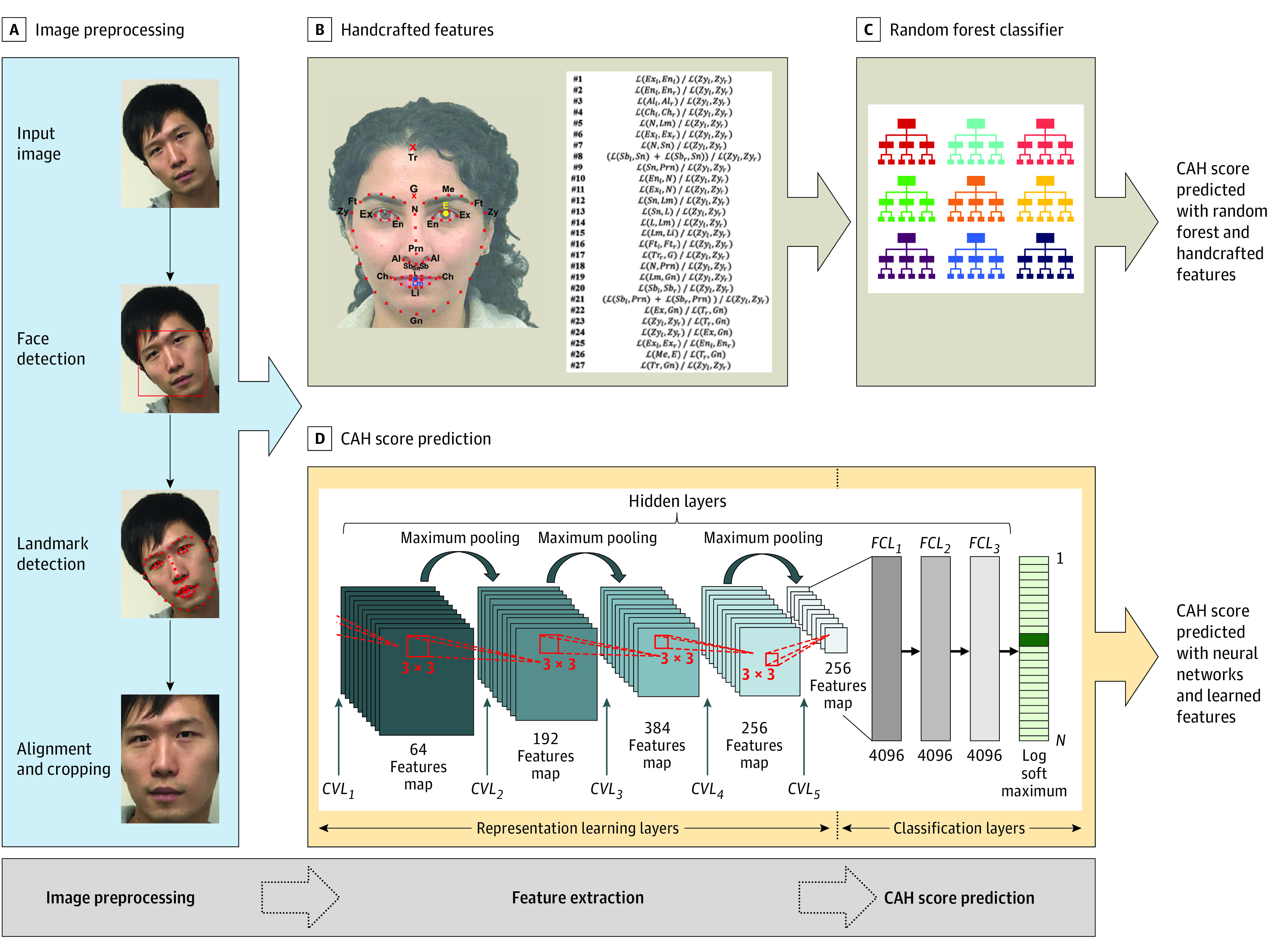
Congenital Adrenal Hyperplasia (CAH) Classification Pipelines Using Handcrafted Features and Learned Representations Illustration of our CAH classification pipelines, including various preprocessing steps of the input image and using both handcrafted features and learned representations. A, The input image was preprocessed by automatically detecting the face region in the image, detecting the locations of the 68 facial landmarks, and aligning and cropping the face region. B, A total of 27 handcrafted features were calculated using the detected landmarks. C, Classical machine learning classifiers, such as random forests, were used to predict the CAH score based on the handcrafted features. D, A deep neural network was used to extract learned representations from the preprocessed image and predict the CAH score without predefined features. CVL indicates convolutional layer; FCL, fully connected layer.

Figure 1 summarizes the approach of the study, including automatic face and facial landmark detection, handcrafted feature extraction, and CAH prediction using both handcrafted and learned representations. For image preprocessing, we applied off-the-shelf techniques for face detection, landmark detection, and alignment and cropping (Figure 1A). We detected the face bounding box and 68 facial landmarks on the input image. The detected landmarks were used to estimate the 3-dimensional (3D) pose of the face (ie, yaw, pitch, and roll rotation angles). We used the yaw angle to decide whether a given image could be included. The yaw angle represents the front of the face. Only face images with a yaw angle less than 30° were considered.^39^ We used the 68 detected landmarks to rotate and perform geometric alignment and cropping of the face to eliminate effects of face pose in subsequent analyses; this strategy has been shown to improve facial analysis tasks.^43^

### Facial Landmarks and Handcrafted Features

We extracted 27 handcrafted features by calculating the 2D Euclidean distances between the 68 landmarks detected on the face (Figure 1B and eFigure 1 in the Supplement).^20,21,23^ These features have been used for the study of sex differences of the face and the association of prenatal androgens with facial morphologic features.^17,20,44^ Because the landmark on top of the forehead is not a standard landmark that is detected by an off-the-shelf method, we manually annotated the entire data set with this landmark. We used these 27 handcrafted features to perform statistical analysis of the discriminability of features between patients with CAH and controls. The details of these landmarks and features are provided in eFigure1 and eFigure 2 in the Supplement.

### Feature Extraction and CAH Score Prediction

We predicted CAH using machine learning methods, which can be generally categorized based on how features are extracted from data into methods that use predefined handcrafted features (Figure 1C) and methods that depend on representation learning in which features are learned from the data (ie, learned representations) using deep neural networks (Figure 1D). We used both techniques to investigate whether facial features differed significantly between patients with CAH and controls.

For techniques based on handcrafted features, such as a support vector machine, we extracted the aforementioned 27 handcrafted features and passed them to linear discriminant analysis and random forest classifiers to predict a CAH score indicating CAH group membership (Figure 1C).^45,46^ Because deep learning–based techniques depend on learning features directly from data, the learned features can either be fed into a classifier or be part of a deep neural network trained end to end.

Therefore, we fed the aligned face image into a convolutional neural network such that the network learned the needed features to predict the CAH score. We used the VGG16 model, which was pretrained to perform a face recognition task using a data set of 3 million face images.^34^ The classification layers of VGG16 were replaced with a small network including 3 fully connected layers with a 2-output sigmoid layer indicating the CAH probability. In VGG16, the dimensionality of learned representations is 4096, which is higher than the 27D feature vector and encodes more information for use in CAH score prediction. eFigure 3 in the Supplement shows example visualization of feature maps of the convolutional layers 1 to 5) (Figure 1D) of our VGG16 model. The feature maps of the deeper layers represent higher-level information, which is harder to interpret compared with earlier layers, which represent low-level features (lines, edges, and orientations) and are easier to interpret.

Because the size of our CAH data set was smaller than the data set used to train VGG16, we froze the weights of the feature extraction part of the network and only trained the last layer of the modified network, exploiting the similarities between the facial recognition domain and CAH facial analysis. This training scheme prevents the network from overfitting on the training data set. The optimization process uses stochastic gradient descent with an initial learning rate of 0.05 using a cross-entropy loss. We trained the network for 20 epochs.

### Training and Testing Protocol of CAH Score Prediction

Owing to the data set size and to avoid overfitting and bias, we adopted a 6-fold cross-validation strategy in which we divided the data into 6 folds of roughly equal sizes; the images of each subject appeared in only 1 fold to ensure statistical independence of all folds. For each experiment, 1 fold was used for testing, 90% of the remaining 5 folds were used for training, and 10% were used for validation. The distribution of CAH and control sample images was approximately the same among the 6 folds (eTable 1 in the Supplement).

### Statistical Analysis

#### Group Differences in Handcrafted Features

To evaluate group differences in 27 handcrafted features (eFigure 2 in the Supplement), we performed 2-tailed *t* tests for analysis of the handcrafted features between the CAH and control groups. We considered a 2-sided *P* < .05 to be statistically significant. Analyses were performed with NumPy and Scipy standard libraries in Python, version 3.7.6.^47^

#### Evaluating CAH Prediction Accuracy

Given an input image to the pipeline, a CAH score was predicted by our models, which took values within 0 and 1 [0,1] representing the probability of a test image being CAH. A predicted CAH score closer to 1 indicated a higher probability of having CAH. These predicted CAH scores were binarized using thresholds varied within [0,1]. A false positive–to-negative ratio and true positive–to-positive ratio were calculated using the binarized decisions and then used to measure the performance of the different CAH prediction techniques in terms of area under curve (AUC) for the receiver operating characteristic curve, which were computed with 95% CIs.^48^

An amalgam was computer-generated by first detecting facial landmarks for all faces in the data set and using these landmarks to align the faces on top of each other by scaling and rotating the images. Aligned faces were then averaged for all females and males within CAH and control groups. These four landmark templates illustrate the differences of facial landmarks between faces of individuals with CAH and faces of controls.

Class activation maps (CAMs) are heat maps indicating regions in the image that the neural network uses to predict the particular category (CAH or control) to which the input image belongs.^49^ These are generated by backpropagating the predicted category through the network to visualize the areas used to produce the prediction.

## Results

The study included 122 individuals with CAH (62 [60.8%] female; mean [SD] age, 11.6 [7.1] years [range, 3 weeks to 29 years), of whom 81 were youths (aged 0-18 years) and 21 were young adults (aged 19 to 29 years); 81 had salt-wasting CAH, and 21 had simple-virilizing CAH. A total of 59 controls (30 [50.8%] female; mean [SD] age, 9.0 [5.2] years [range, 3 weeks to 26 years]) were recruited from the clinic (Table). We acquired 993 CAH sample images and 446 control sample images. Among patients with CAH, 60 of 102 (59%) were Hispanic, and among controls, 34 of 59 (57.6%) were Hispanic. We studied 85 additional controls (48 [60%] female) younger than 29 years (1078 sample images) selected from public data sets. The Table summarizes the study population characteristics.

**Table.** Study Population by Sex and Group

Group	Female, No.		Male, No.		Total, No.	
	Persons	Samples	Persons	Samples	Persons	Samples
CAH	62	618	40	375	102	993
Control^a^						
Clinic	30	206	29	240	59	446
Data sets	51	696	34	382	85	1078
Total	143	1520	103	997	246	2517

Abbreviation: CAH, congenital adrenal hyperplasia.

^a^ Controls included participants tested at the clinic and control data selected from publicly available data sets.^39,40,41^

### Group Differences in Handcrafted Features

Comparing 27 handcrafted facial features (used for the study of sex differences of the face and the association of prenatal androgens with facial morphologic features) between patients with CAH and controls, we found that 11 of 27 facial features were statistically different between the groups. *P* values are reported in eTable 2 in the Supplement.

### Accuracy of CAH Prediction

**Figure 2.**
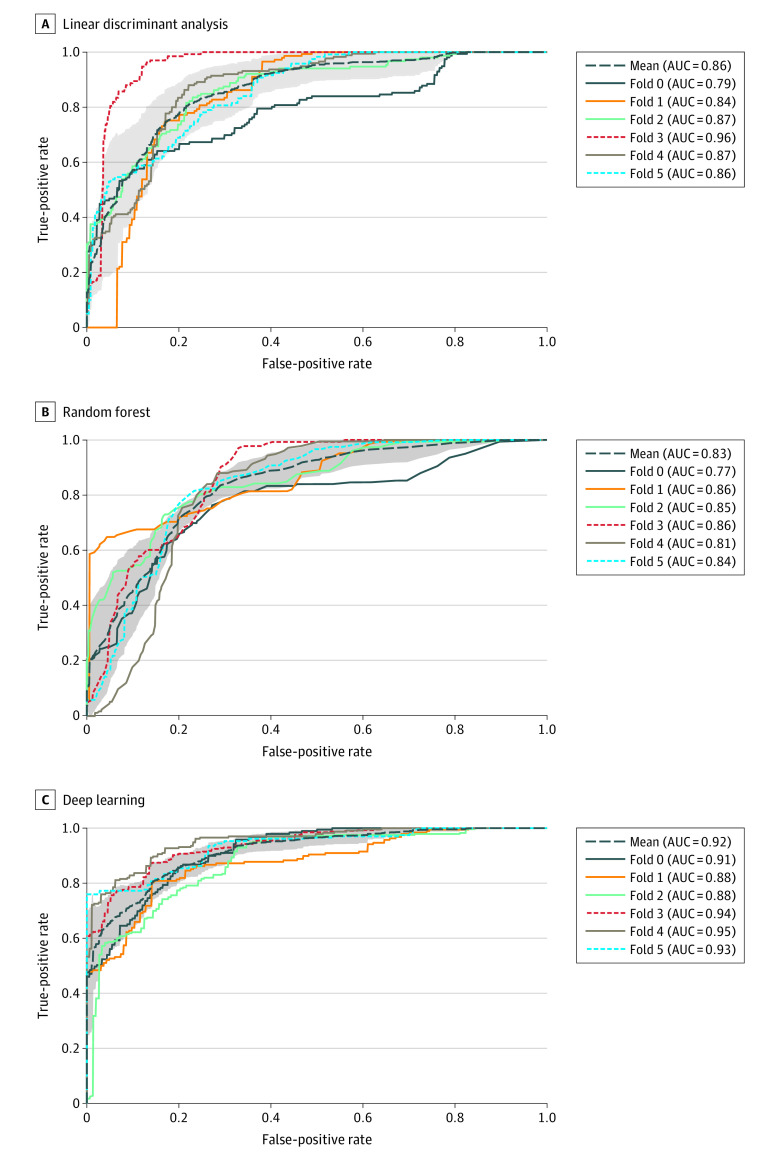
Performance Analysis of Congenital Adrenal Hyperplasia (CAH) Scoring Using Machine Learning Techniques Receiver operating characteristic curves are shown for each method over 6 folds as well as the mean area under the curve (AUC). Shaded areas indicate SDs.

The receiver operating characteristic curves for the 6-fold partitioning for CAH classification using 27 handcrafted features from linear discrimination analysis and random forest classifiers are shown in Figure 2A and B, respectively. We obtained a mean (SD) AUC of 86% (5%) using linear discrimination analysis and a mean (SD) AUC of 83% (3%) using random forest classifiers by calculating the mean AUCs of the 6 folds; this method indicates the ability to differentiate between the features of patients with CAH and controls. Extracting features using VGG16 provided a high prediction accuracy, with a mean (SD) AUC of 92% (3%) by determining the mean of the 6 folds (Figure 2C), thus demonstrating the presence of recognizable facial features that differed between patients with CAH and controls.

Among patients with CAH, the mean (SD) CAH score was similar between Hispanic (0.82 [0.28]) and non-Hispanic (0.81 [0.30]) patients (*P* = .80). The mean (SD) CAH score was also similar between patients with a Tanner stage of I to II (n = 52; 0.83 [0.28]) and those with a Tanner stage of III to V (n = 50; 0.81 [0.30]) (*P* = .96). There were no significant differences between the youngest patients (0-6 years; n = 31; mean [SD] score, 0.88 [0.24]) and those aged 7 to 12 years (n = 26; mean [SD] score, 0.76 [0.32]; *P* = .11), 13 to 18 years (n = 29; mean [SD] score, 0.82 [0.31]; *P* = .64), and 19 to 29 years (n = 16; mean [SD] score, 0.85 [0.28]; *P* = .94).

### Explanation of CAH Prediction

**Figure 3.**
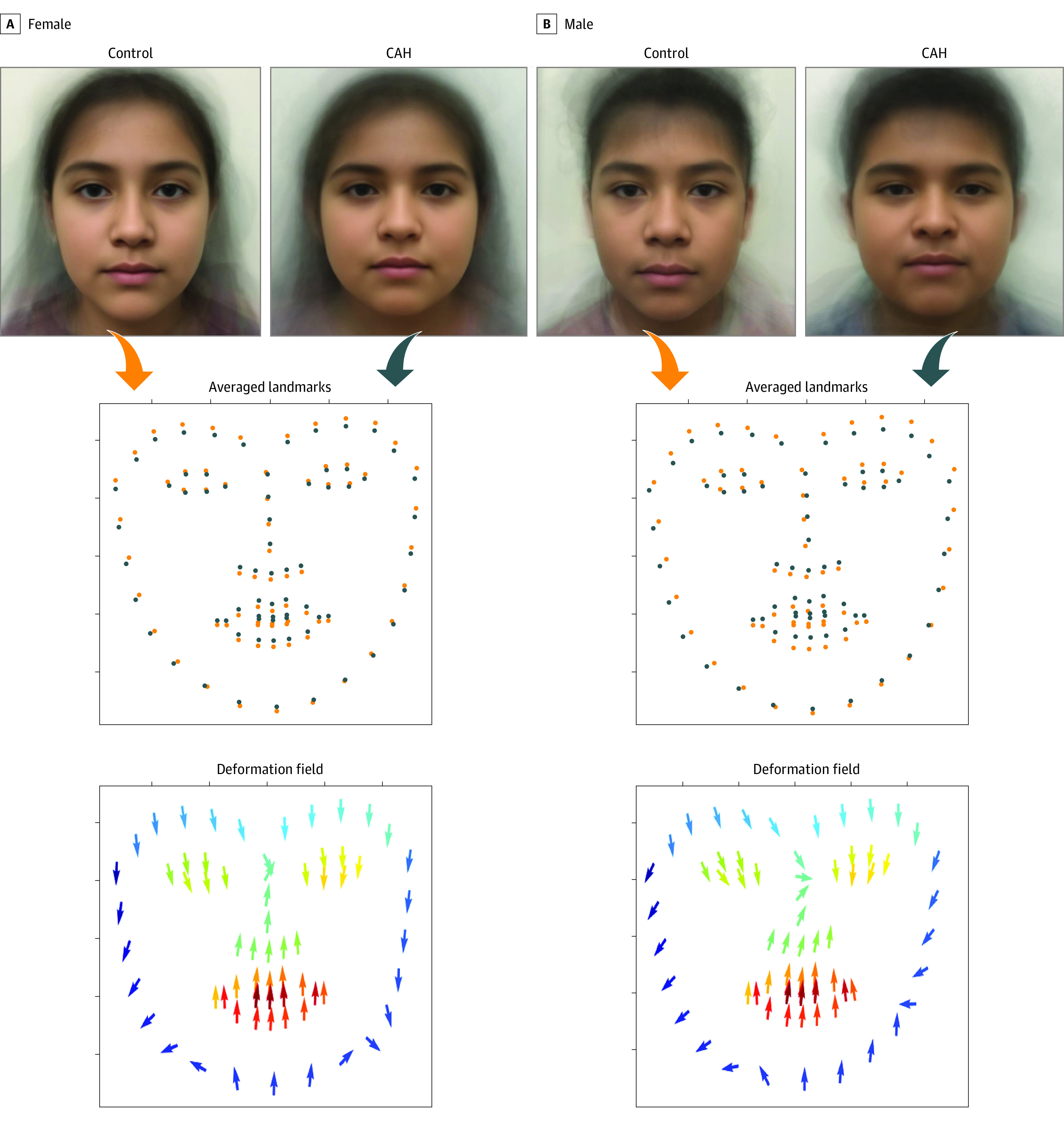
Facial Landmark Templates of Averaged Facial Images in Patients With Congenital Adrenal Hyperplasia (CAH) and Control Individuals Top, The computer-generated averaged amalgam faces of patients with CAH and controls by sex are shown. The second row visualizes the overlaid 68 facial landmarks of the control group (orange) and the group with CAH (blue). The bottom row visualizes the deformation field introduced by CAH, with the direction of the arrows moving from facial landmarks of controls to those of patients with CAH. This deformation field helps interpret the averaged facial images.

We examined the computer-generated amalgam face image of 1 female and 1 male per group (CAH and control). We found on deformation analysis that there was deviation of facial landmarks in patients with CAH compared with sex-matched controls (Figure 3).

**Figure 4.**
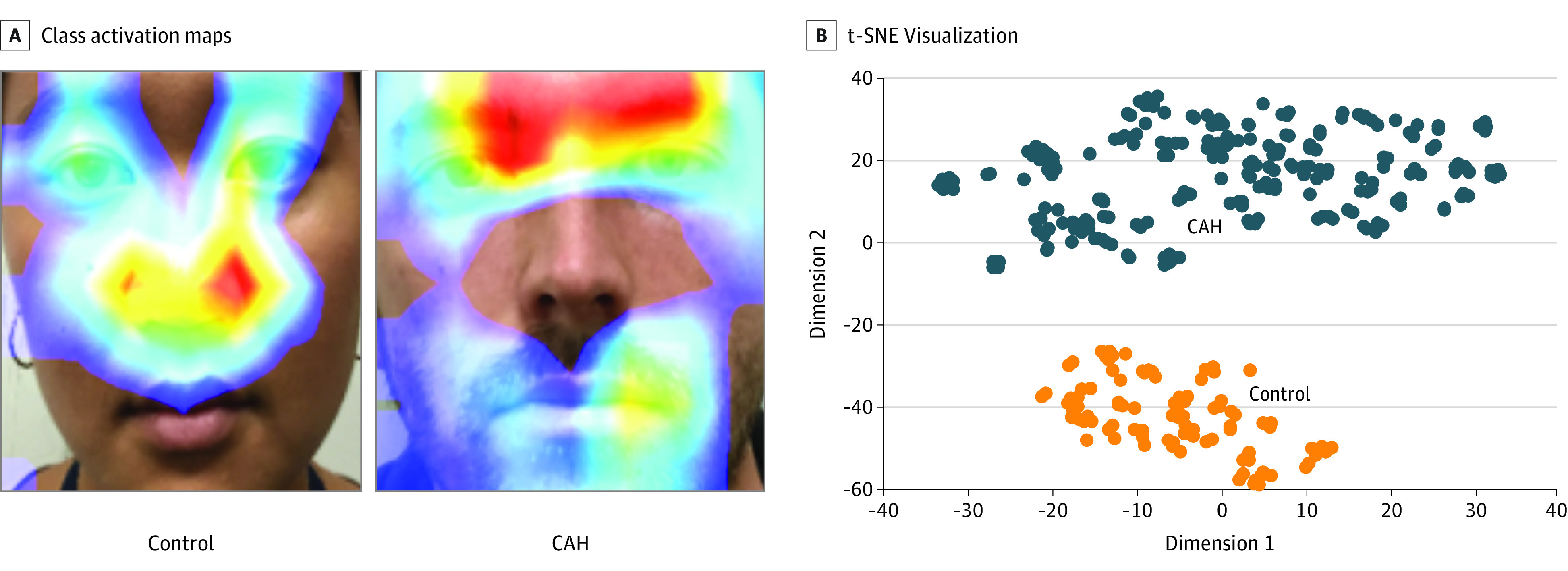
Class Activation Maps and t-Distributed Stochastic Neighbor Embedding (t-SNE) Visualization A, Red areas indicate the more contributory regions to the final predicted congenital adrenal hyperplasia (CAH) score. B, Visualization of the class activation maps for patients with CAH and controls.

For both CAH and control groups, we generated CAMs (Figure 4A).^49^ A 2D t-distributed stochastic neighbor embedding visualization (Figure 4B) of CAMs for all individuals further showed that the CAH and control groups were completely separable using deep learning, explaining the prediction accuracy.^50^

We also performed regionwise analysis of the deep neural network pipeline to study the importance of 5 different regions for prediction of CAH score (eFigure 4 in the Supplement). We blocked 1 region at a time to assess performance degradation with each blocked region that was not passed to the neural network. A lower AUC signifies a greater impact of hiding the region. The ranking of the 5 facial regions from high to low importance was nose region, upper face region, lower face region, mouth region, and region around the eyes. The 2 facial regions with the highest importance, the nose region and the upper face region, were also the most contributory on CAMs.

## Discussion

In this study, machine learning was used to study facial morphologic features that predict severe, classical CAH due to 21-hydroxylase deficiency. Patients with CAH were reliably distinguished from healthy controls through CAH scores. The most accurate prediction was obtained by using a deep learning–based technique (eg, VGG13 model and learned representations of input images), with a 92% AUC compared with classical machine learning methods (eg, linear discrimination analysis and random forest classifiers, and handcrafted features). To our knowledge, differences in morphological facial features have not yet been reported in individuals with CAH. The predictive power of facial morphology in our data set shows the utility of deep learning methods to detect more subtle facial features in patients with CAH.

We used multiple methods to explain the differences in facial morphologic features between CAH and control groups. We found a deviation of facial features between the groups by using deformation fields generated from facial landmark templates. We observed a tendency for deformation fields to point to the center of the face, which is worthy of further investigation. In addition, our analyses derived from deep learning of facial regions found the nose and upper face regions to be most contributory in this data set.

Our subanalysis of 27 handcrafted facial features related to sexual dimorphism and prenatal testosterone found that 11 features were significantly different between patients with CAH and controls. This may represent prenatal organizational and/or postnatal activational effects of androgen excess on facial morphologic features in patients with CAH. Among patients with CAH, there were no differences in CAH score by either age or stage of puberty. A combination of organizational and activational effects of excess androgens likely determines whether an individual with CAH develops 1 or more of these adverse outcomes over their lifespan,^51,52,53^ and machine learning of facial features could be used longitudinally as a phenotypic biomarker to better understand the effects of androgen excess in the population with CAH. There is otherwise a paucity of biomarkers of fetal testosterone exposure because amniotic fluid sampling remains impractical and the second-to-fourth digit ratio as an indirect marker may need to be interpreted with caution if extrapolated to correlations with postnatal behavior.^54^ Machine learning of facial morphologic features is already being applied longitudinally to better understand aging in humans, with the creation of markers of aging that can be studied over a lifetime.^55^ The potential role of both androgen excess and cortisol deficiency needs to be further explored in the development of distinct facial features in patients with classical CAH.

### Limitations

This study has limitations. We studied a relatively small sample size of patients with CAH. Larger, multicenter studies are needed to increase the sample size of patients with CAH. In addition, we used 2D images of the face for predicting CAH, which do not provide as much facial information as 3D images that are collected using infrared cameras or stereo photogrammetric systems. The next steps involve building on the current work with 3D facial models in patients with CAH to describe exact morphologic feature differences in detail, similar to a study of fetal alcohol syndrome.^19^. Furthermore, although our results indicate that a better CAH prediction was achieved by deep learning methods, a common criticism of deep learning is the lack of interpretability. More sophisticated methods, such as attention maps and face parsing, need to be investigated to explain the findings of the deep learning models. In addition, there is a large Hispanic population in Los Angeles and a low incidence of CAH in African American and Asian individuals; thus, achieving racial/ethnic diversity in this study was challenging. Although the majority of the study population was Hispanic, there was no difference in predicted CAH score between Hispanic and non-Hispanic patients with CAH.

## Conclusions

In this cross-sectional study, with use of machine learning approaches to study facial morphologic features in patients with CAH, we found that facial features distinguished these patients from unaffected, healthy controls, with a high ability to predict CAH. Our findings highlight the potential for deep learning to uncover morphologic differences in patients with more subtle features. Facial features as a phenotypic biomarker could be studied from birth or before birth if possible to broaden understanding of the clinical phenotype and adverse clinical outcomes. Further study is merited to understand the etiology of affected facial morphologic features in patients with CAH as well as associations with disease severity.

## Notes

Supplement. **eFigure 1.** Visualization of the 68 facial landmarks considered in our facial analysis **eFigure 2.** Definitions of the 27 handcrafted features considered in our facial analysis **eTable 1.** Data distribution per fold of 6-fold partitioning strategy for CAH and controls **eTable 2.** Euclidean features and measurements in CAH versus controls **eFigure 3.** Feature maps of the VGG16 model layers in the deep neural network **eFigure 4.** Regionwise facial analysis of different regions (upper face, lower face, region around eyes, region around nose, and region around mouth)
