# Supplementary material for: Assessment of Facial Morphologic Features in Patients With Congenital Adrenal Hyperplasia Using Deep Learning
Source: JAMA Netw Open. 2020 Nov 18;3(11):e2022199. doi: 10.1001/jamanetworkopen.2020.22199 (PMC7675110; doi:10.1001/jamanetworkopen.2020.22199)
Supplement: Supplement. — eFigure 1. Visualization of the 68 facial landmarks considered in our facial analysis eFigure 2. Definitions of the 27 handcrafted features considered in our facial analysis eTable 1. Data distribution per fold of 6-fold partitioning strategy for CAH and controls eTable 2. Euclidean features and measurements in CAH versus controls eFigure 3. Feature maps of the VGG16 model layers in the deep neural network eFigure 4. Regionwise facial analysis of different regions (upper face, lower face, region around eyes, region around nose, and region around mouth) [file jamanetwopen-e2022199-s001.pdf]

Supplemental Online Content

AbdAlmageed W, Mirzaalian H, Guo X, et al. Assessment of facial morphologic features in patients with congenital adrenal hyperplasia using deep learning. *JAMA Netw Open*. 2020;3(11):e2022199.
doi:10.1001/jamanetworkopen.2020.22199

eFigure 1. Visualization of the 68 facial landmarks considered in our facial analysis
eFigure 2. Definitions of the 27 handcrafted features considered in our facial analysis
eTable 1. Data distribution per fold of 6-fold partitioning strategy for CAH and controls
eTable 2. Euclidean features and measurements in CAH versus controls
eFigure 3. Feature maps of the VGG16 model layers in the deep neural network
eFigure 4. Regionwise facial analysis of different regions (upper face, lower face, region around eyes, region around nose, and region around mouth)

This supplemental material has been provided by the authors to give readers additional information about their work.

© 2020 AbdAlmageed W et al. *JAMA Network Open.*

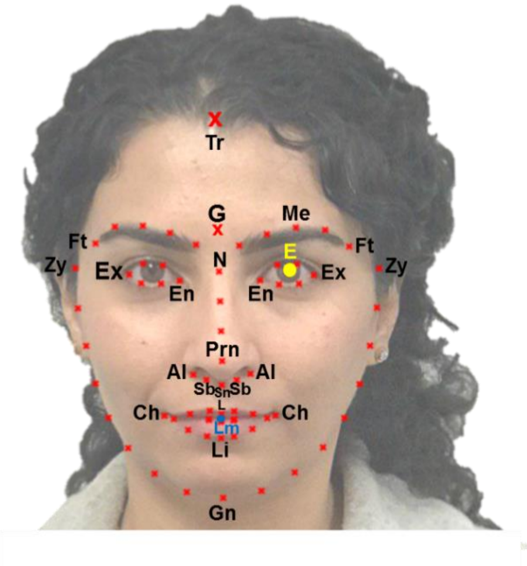

Landmark	Name	Definition
G	Glabella	The most convex sagittal midline point between the eyebrows
Ft	Frontotemporale	The point of concavity on each side of the forehead above the supraorbital rim, lateral to the elevation of the linea temporalis
Gn	Gnathion	The lowest median landmark on the inferior aspect of the mandible
En	Endocanthion	The most medial point of the palpebral fissure, at the inner commissure of the eye
Ex	Exocanthion	The most lateral point of the palpebral fissure, at the outer commissure of the eye
N	Nasion	The sagittal midline point of the nasal root at the nasofrontal suture
Al	Alare	The most lateral extents of the alar contours
Prn	Pronasale	The most protrusive point of the nasal tip (apex nasi)
Ch	Cheilion	The most lateral points at the labial commissure
L	Labiale superius	The midline point of the upper lip (at the upper vermilion line)
Li	Labiale inferius	The midline point of the lower lip (at the lower vermilion line)
Zy	Zygion	The most lateral extents of the zygomatic arches
Sn	Subnasal	The lowest point of the nose, above the lip
Sb		Midpoint between Al and Sn
Lm		Cross-section of upper and lower lip with horizontal line of the lip
E		Midpoint of the eye
Me		Midpoint of the eyebrow
Tr		Top of the forehead

eFigure 1. Visualization of 68 facial landmarks considered in our facial analysis.

© 2020 AbdAlmageed W et al. *JAMA Network Open.*

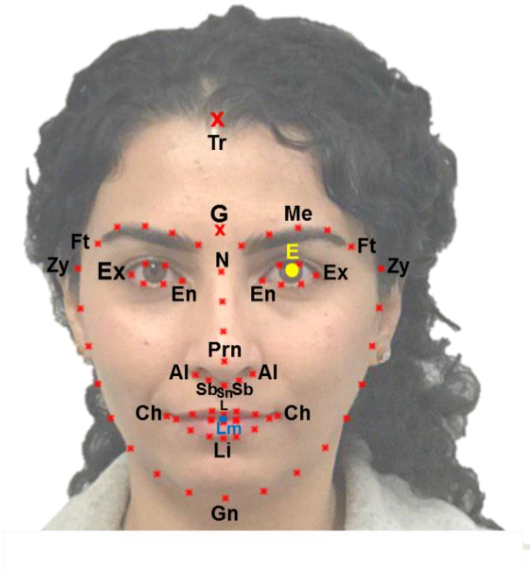

Feature #	Feature description	Feature #	Feature description
#1	$\mathcal{L}(Ex_l,\ En_l)\ /\ \mathcal{L}(Zy_l, Zy_r)$	#15	$\mathcal{L}(Lm,\ Li)\ /\ \mathcal{L}(Zy_l, Zy_r)$
#2	$\mathcal{L}(En_l,\ En_r)\ /\ \mathcal{L}(Zy_l, Zy_r)$	#16	$\mathcal{L}(Ft_l,\ Ft_r)\ /\ \mathcal{L}(Zy_l, Zy_r)$
#3	$\mathcal{L}(Al_l,\ Al_r)\ /\ \mathcal{L}(Zy_l, Zy_r)$	#17	$\mathcal{L}(Tr_r,\ G)\ /\ \mathcal{L}(Zy_l, Zy_r)$
#4	$\mathcal{L}(\mathrm{Ch}_l,\ \mathrm{Ch}_r)\ /\ \mathcal{L}(Zy_l, Zy_r)$	#18	$\mathcal{L}(N,\ Prn)\ /\ \mathcal{L}(Zy_l, Zy_r)$
#5	$\mathcal{L}(N,\ Lm)\ /\ \mathcal{L}(Zy_l, Zy_r)$	#19	$\mathcal{L}(Lm,\ Gn)\ /\ \mathcal{L}(Zy_l, Zy_r)$
#6	$\mathcal{L}(Ex_l,\ Ex_r)\ /\ \mathcal{L}(Zy_l, Zy_r)$	#20	$\mathcal{L}(Sb_l,\ Sb_r)\ /\ \mathcal{L}(Zy_l, Zy_r)$
#7	$\mathcal{L}(N,\ Sn)\ /\ \mathcal{L}(Zy_l, Zy_r)$	#21	$(\mathcal{L}(Sb_l,\ Prn)\ +\ \mathcal{L}(Sb_r,\ Prn)\ )\ /\ \mathcal{L}(Zy_l, Zy_r)$
#8	$(\mathcal{L}(Sb_l,\ Sn)\ +\ \mathcal{L}(Sb_r,\ Sn))\ /\ \mathcal{L}(Zy_l, Zy_r)$	#22	$\mathcal{L}(Ex,\ Gn)\ /\ \mathcal{L}(T_r, Gn)$
#9	$\mathcal{L}(Sn,\ Prn)\ /\ \mathcal{L}(Zy_l, Zy_r)$	#23	$\mathcal{L}(Zy_l, Zy_r)\ /\ \mathcal{L}(T_r, Gn)$
#10	$\mathcal{L}(En_l,\ N)\ /\ \mathcal{L}(Zy_l, Zy_r)$	#24	$\mathcal{L}(Zy_l, Zy_r)\ /\ \mathcal{L}(Ex, Gn)$
#11	$\mathcal{L}(Ex_l,\ N)\ /\ \mathcal{L}(Zy_l, Zy_r)$	#25	$\mathcal{L}(Ex_l,\ Ex_r)\ /\ \mathcal{L}(En_l, En_r)$
#12	$\mathcal{L}(Sn,\ Lm)\ /\ \mathcal{L}(Zy_l, Zy_r)$	#26	$\mathcal{L}(Me,\ E)\ /\ \mathcal{L}(T_r, Gn)$
#13	$\mathcal{L}(Sn,\ L)\ /\ \mathcal{L}(Zy_l, Zy_r)$	#27	$\mathcal{L}(Tr,\ Gn)\ /\ \mathcal{L}(Zy_l, Zy_r)$
#14	$\mathcal{L}(L, Lm)\ /\ \mathcal{L}(Zy_l, Zy_r)$		

eFigure 2. Definitions of 27 handcrafted features considered in our facial analysis.

© 2020 AbdAlmageed W et al. *JAMA Network Open.*

eTable 1. Data distribution per fold of 6-fold partitioning strategy for CAH and controls

Fold	CONTROL Subject	CONTROL Sample	CONTROL* Subject	CONTROL* Sample	CAH Subject	CAH Sample
Fold#0	10	72	14	117	17	182
Fold#1	10	66	14	122	17	164
Fold#2	10	71	14	212	17	153
Fold#3	10	91	14	307	17	155
Fold#4	10	97	14	136	17	195
Fold#5	9	49	15	184	17	144

Number of unique subjects and total number of sample images per fold
Controls include participants tested in clinic (CONTROL) as well as control data selected from publicly available datasets (CONTROL*).

© 2020 AbdAlmageed W et al. *JAMA Network Open.*

eTable 2. Euclidean features and measurements in CAH versus controls

Feature#	Feature description	P
#1	$\mathcal{L}(Ex_l,\ En_l)\ /\ \mathcal{L}(Zy_l, Zy_r)$	0.000
#2	$\mathcal{L}(En_l,\ En_r)\ /\ \mathcal{L}(Zy_l, Zy_r)$	0.545
#3	$\mathcal{L}(Al_l,\ Al_r)\ /\ \mathcal{L}(Zy_l, Zy_r)$	0.044
#4	$\mathcal{L}(\mathrm{Ch}_l,\ \mathrm{Ch}_r)\ /\ \mathcal{L}(Zy_l, Zy_r)$	0.311
#5	$\mathcal{L}(N,\ Lm)\ /\ \mathcal{L}(Zy_l, Zy_r)$	0.000
#6	$\mathcal{L}(Ex_l,\ Ex_r)\ /\ \mathcal{L}(Zy_l, Zy_r)$	0.008
#7	$\mathcal{L}(N,\ Sn)\ /\ \mathcal{L}(Zy_l, Zy_r)$	0.000
#8	$(\mathcal{L}(Sb_l,\ Sn)\ +\ \mathcal{L}(Sb_r,\ Sn))\ /\ \mathcal{L}(Zy_l, Zy_r)$	0.113
#9	$\mathcal{L}(Sn,\ Prn)\ /\ \mathcal{L}(Zy_l, Zy_r)$	0.485
#10	$\mathcal{L}(En_l,\ N)\ /\ \mathcal{L}(Zy_l, Zy_r)$	0.055
#11	$\mathcal{L}(Ex_l,\ N)\ /\ \mathcal{L}(Zy_l, Zy_r)$	0.002
#12	$\mathcal{L}(Sn,\ Lm)\ /\ \mathcal{L}(Zy_l, Zy_r)$	0.082
#13	$\mathcal{L}(Sn,\ L)\ /\ \mathcal{L}(Zy_l, Zy_r)$	0.060
#14	$\mathcal{L}(L, Lm)\ /\ \mathcal{L}(Zy_l, Zy_r)$	0.695
#15	$\mathcal{L}(Lm,\ Li)\ /\ \mathcal{L}(Zy_l, Zy_r)$	0.189
#16	$\mathcal{L}(Ft_l,\ Ft_r)\ /\ \mathcal{L}(Zy_l, Zy_r)$	0.010
#17	$\mathcal{L}(Tr_r,\ G)\ /\ \mathcal{L}(Zy_l, Zy_r)$	0.126
#18	$\mathcal{L}(N,\ Prn)\ /\ \mathcal{L}(Zy_l, Zy_r)$	0.000
#19	$\mathcal{L}(Lm,\ Gn)\ /\ \mathcal{L}(Zy_l, Zy_r)$	0.077
#20	$\mathcal{L}(Sb_l,\ Sb_r)\ /\ \mathcal{L}(Zy_l, Zy_r)$	0.107
#21	$(\mathcal{L}(Sb_l,\ Prn)\ +\ \mathcal{L}(Sb_r,\ Prn)\ )\ /\ \mathcal{L}(Zy_l, Zy_r)$	0.703
#22	$\mathcal{L}(Ex,\ Gn)\ /\ \mathcal{L}(T_r, Gn)$	0.127
#23	$\mathcal{L}(Zy_l, Zy_r)\ /\ \mathcal{L}(T_r, Gn)$	0.001
#24	$\mathcal{L}(Zy_l, Zy_r)\ /\ \mathcal{L}(Ex, Gn)$	0.025
#25	$\mathcal{L}(Ex_l,\ Ex_r)\ /\ \mathcal{L}(En_l, En_r)$	0.033
#26	$\mathcal{L}(Me,\ E)\ /\ \mathcal{L}(T_r, Gn)$	0.347
#27	$\mathcal{L}(Tr,\ Gn)\ /\ \mathcal{L}(Zy_l, Zy_r)$	0.117

© 2020 AbdAlmageed W et al. *JAMA Network Open.*

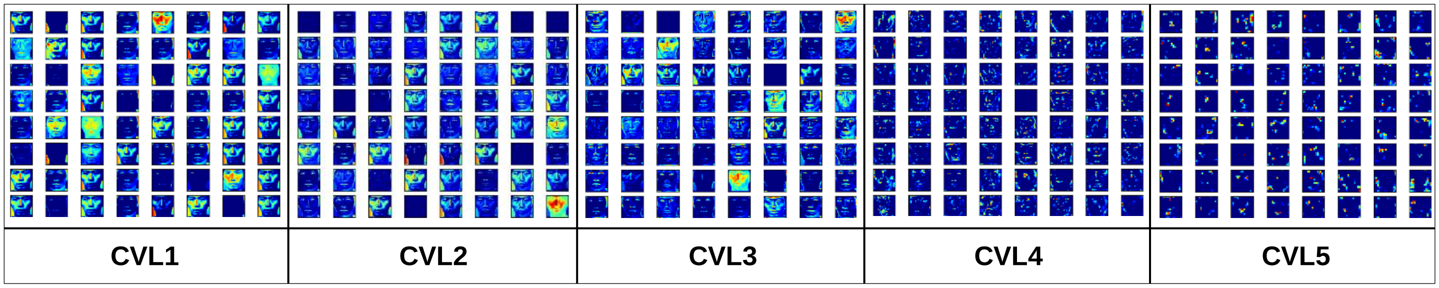

eFigure 3. Feature maps of the VGG16 model layers (CVL1, CVL2, CVL3, CVL4, and CVL5 depicted in Figure 1). It can be seen that as we move from earlier layers to the deeper ones (CVL1 to CVL5), the features are moved from low level features (like edges) to high level features, and high level features are not easy to interpret.

© 2020 AbdAlmageed W et al. *JAMA Network Open.*

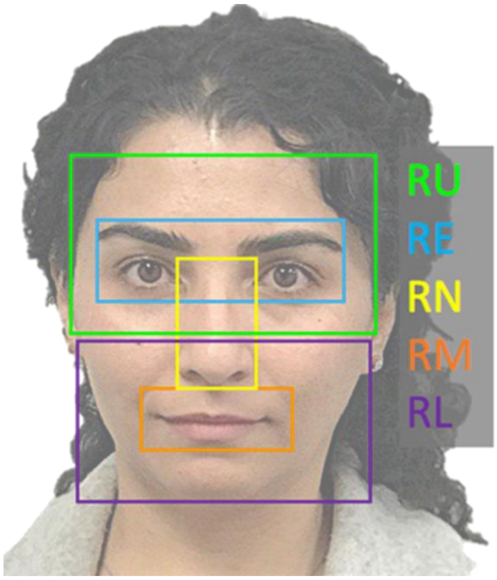

	AUC
**RU**	0.71 ± 0.13
**RL**	0.79 ± 0.14
**RN**	0.69 ± 0.17
**RE**	0.82 ± 0.09
**RM**	0.80 ± 0.14

eFigure 4. Regionwise facial analysis of five different regions: upper face region (RU), lower face region (RL), region around eyes (RE), region around nose (RN), and region around mouth (RM). To find the importance of each facial region, we blocked one region at a time to assess the drop in performance with each blocked region that was not passed to the neural network. The area under the curve (AUC) is listed (mean ± SD) per region, with a lower AUC signifying a greater impact of hiding the region.

© 2020 AbdAlmageed W et al. *JAMA Network Open.*
